# Cardiovascular Rehabilitation in Coronary Artery Disease and Better Knowledge of Its Own Disease

**DOI:** 10.5935/abc.20180258

**Published:** 2019-01

**Authors:** Luiz Antonio Machado César

**Affiliations:** Faculdade de Medicina da Universidade de São Paulo, São Paulo, SP - Brazil; Instituto do Coração do Hospital das Clínicas da Faculdade de Medicina da Universidade de São Paulo, São Paulo, SP - Brazil

**Keywords:** Coronary Artery Disease/rehabilitation, Health Promotion, Preventive Medicine, Cardiac Rehabilitation, Exercise, Patient Health Questionnaire

In the last ten to fifteen years, we are seen more and more concern with patients´ awareness about their diseases.^1^^-^^3^ Campaigns have been brought to the public to teach them about symptoms and signs that might bring concern and make them ask for help and go to an emergency room or, in the United States to call 911. This is true especially for patients with coronary artery disease (CAD), given the possibility of better quality of life and in some cases even a better prognosis of the disease in respect to morbidity and mortality.

Beside this kind of campaigns, recently others started to aware people already with a diagnosis of CAD to the symptoms that may alert them of a problem and the consciousness about all medicines that modify the course of the disease and ways of life that are proved to contribute to this amelioration, as is physical activity.^4^^-^^8^

In this issue, dos Santos et al.^9^ did validate a questionnaire to evaluate patients with CAD and on cardiovascular rehabilitation (rehab) programs in order to assess their knowledge about their own disease. Interestingly they first validate a previous (CADE-Q) questionnaire in Portuguese, then validated an English translated version. After that, they do construct a CAD-Q II questionnaire, but in English. The motivation to do this second version was that some questions should be better structured to the understanding of the patients and a psychosocial approach might be a part of it. After publishing it in the English language then they decide to translate to the Portuguese language. It is really a different approach going from one language to another and coming back to the first one, what is not usually done. The need for implementing the CADE-Q with other components was based on cardiac rehabilitation programs focused on patients with CAD. The objective of this paper is to validate the English version of CAD-Q II. The job was done according to the available tests used to validate questionnaires from one language to other, using Cronbach's alpha test. What they found called attention, although already known from other studies with questionnaires.

As higher the intellectual level of participants or their family income, better are they know of their disease, as shown in this validation. As the authors comment at discussion, they applied the questionnaire asking the questions, rather than applying a self-questionnaire as was done in the validation of the English version. Someone can think of this being a consequence of the intellectual level of our people. If this is true, it may be really an inadequacy for this questionnaire to be applied and it will be of importance to test more times to make sure it is truthful to be generally applied elsewhere. This does not invalidate this questionnaire, on the contrary, it can be otherwise be retested and then confirm confidence and feasibility.

We know the importance of awareness of people already with CAD, in this case specifically to patients on cardiac rehab, for symptoms that can be a warning for acute coronary events. But a questionnaire first done in the Portuguese language would be better validated if the second version had been also done in Portuguese and not a change to English, plus validation in that language and then a way back to the Portuguese language with cultural adjustments needed in the first and the second paths for validation. Besides it is important to understand, many times we treat patients with CAD and do not be aware of the costs/benefits of its treatment and many times, after years of observation, we do not find any difference between treatments and strategies^8^ to the most frequent and first killer in the world. Cardiac rehab is known to reduce mortality in those with CAD, at least in a medium period of follow-up what probably justify a positive cost/effectiveness of these programs. Otherwise, we need longer follow-up, seven to ten years, studies to assure that really cardiac rehab programs are cost/effective.
